# The chemical composition and the preservative, antimicrobial, and antioxidant effects of *Thymus broussonetii* Boiss. essential oil: an *in vitro* and *in silico* approach

**DOI:** 10.3389/fchem.2024.1402310

**Published:** 2024-07-04

**Authors:** Smahane Boukhira, Fatima Ez-Zahra Amrati, Mohamed Chebaibi, Andriy Grafov, Ramzi A. Mothana, Hanan M. Al-Yousef, Dalila Bousta

**Affiliations:** ^1^ Ministry of Health and Social Protection, Higher Institute of Nursing Professions and Health Techniques, Guelmim, Morocco; ^2^ National Agency of Medicinal and Aromatic Plants, Taounate, Morocco; ^3^ Laboratory of Cell Biology and Molecular Genetics (LBCGM), Department of Biology, Faculty of Sciences, Faculty of Sciences, Ibn Zohr University, Agadir, Souss-Massa, Morocco; ^4^ Ministry of Health and Social Protection, Higher Institute of Nursing Professions and Health Techniques, Fez, Morocco; ^5^ Department of Chemistry, University of Helsinki, Helsinki, Finland; ^6^ Department of Pharmacognosy, College of Pharmacy, King Saud University, Riyadh, Saudi Arabia; ^7^ Laboratory of Biotechnology, Health, Agrofood and Environment (LBEAS), Faculty of Sciences Dhar El Mehraz, Sidi Mohamed Ben Abdellah University, Fez, Morocco

**Keywords:** Thymus broussonetii Boiss. essential oil, antioxidant, antimicrobial, natural preservative, molecular docking, gas chromatography/mass spectrometry

## Abstract

**Introduction:**

The aim of this study was to evaluate the antioxidant, antimicrobial, and preservative efficacy of *Thymus broussonetii* Boiss. essential oil (EO) in a topically applied formulation using a challenge test.

**Methods:**

The essential oil was extracted from the aerial part of *T. broussonetii* using hydrodistillation, and the obtained EO was further analyzed by gas chromatography/mass spectrometry (GC/MS). The antioxidant effect of the EO was evaluated using three methods: the inhibition of free radical 2,2-diphenyl-1-picrylhydrazyl (DPPH), β-carotene–linoleic acid, and the ferric reducing antioxidant power (FRAP) methods. The antimicrobial activity and the minimum inhibitory concentration (MIC) of this EO were assayed by the disk-diffusion method and the broth microdilution method, respectively. The preservative efficacy of *T. broussonetii* EO was assayed at 1% and 2% (v/w) in a topical cream formulation using a challenge test against standard-specific microorganisms recommended by the European Pharmacopoeia. Furthermore, the identified phytochemical compounds were docked for their effect on nicotinamide adenine dinucleotide phosphate oxidase, human casein kinase 1 alpha 1 (CSNK1A1), glycogen synthase kinase 3, *Staphylococcus aureus* nucleoside diphosphate kinase, *Escherichia coli* beta-ketoacyl-[acyl-carrier protein] synthase, *Pseudomonas aeruginosa* LasR ligand-binding domain, and sterol 14-alpha demethylase (CYP51) from *Candida albicans*. The ADME/toxicity was predicted by analyzing the absorption, distribution, metabolism, and excretion parameters.

**Results and discussion:**

chemical composition of the EO revealed the presence of thymol (63.09%), p-cymene (11%), and γ-terpinene (8.99%) as the major components. The antioxidant assays revealed that the essential oil exhibited strong antioxidant activity, as indicated by the minimum inhibitory concentration IC_50_ (IC_50_ = 210 ± 0.3 μg/mL for the DPPH assay, IC_50_ = 145 ± 0.1 μg/mL for the β-carotene assay, and IC_50_ = 84 ± 0.21 μg/mL for the FRAP assay) when compared to quercetin and butylated hydroxytoluene (BHT) as controls. The investigated essential oil exhibited important antimicrobial activity against all the tested microorganisms, and the MICs of the EO against bacteria and fungi were 0.02%–1%. Moreover, the EO of *T. broussonetii* evaluated at 2% (v/w) in a cream formulation succeeded in satisfying the A criteria for preservation efficacy against *S. aureus*, *E. coli*, and *Aspergillus brasiliensis* but exhibited less efficacy against *P. aeruginosa* (1.78 log reduction in the number of CFU/g after 7 days of evaluation) and *C. albicans* (1.09 log reduction in the number of CFU/g after 14 days of evaluation) when compared to the synthetic preservative phenoxyethanol 1% (v/w). *In silico* results showed that the antimicrobial activity of *T. broussonetii* EO is mostly attributed to thymol, terpinen-4-ol, and aromadendrene, while the antioxidant activity is attributed to thymol. These results indicate that the EO of *T. broussonetii* possesses important antimicrobial and antioxidant properties and can, therefore, be used as a natural preservative ingredient in the cosmetic industry.

## 1 Introduction

The global market of food, cosmetics, and pharmaceutical products requires continuous tracking of harmful ingredients and microbial contamination for the sake of the safety of both the products and consumers, as these products greatly dominate the consumer’s health, directly or indirectly. Microbial contamination can lead to product degradation, constitute a risk to the health of the consumer, and potentially spread infection. For these reasons, the microbiological safety of pharmaceutical and cosmetic products must be of special interest to the industries (Hye et al., 2020). Preservatives are substances used to limit microbial growth and survival in cosmetic and pharmaceutical products in order to prolong their shelf life (Nowak-Lange et al., 2022). In recent years, there has been considerable interest in the search for natural antimicrobial alternatives, such as essential oils, to avoid the use of chemical agents (Johnson et al., 2000; Seo et al., 2002). Several studies have shown that thyme oils possess antimicrobial properties, with those of the phenol compound type being the most active (Labiad et al., 2022; Pinto et al., 2006; Saad et al., 2010; El Bouzidi et al., 2013). Nowadays, the use of synthetic preservatives has become common and has not been widely accepted by consumers, as they are aware that exposure to preservatives can lead to adverse effects on health, which is a major area of concern for researchers.

Chemicals with antimicrobial properties used as ingredients in cosmetics ensure their durability and safety. Polyphenolic compounds, peptides, essential oils, and plant extracts characterized by these properties are natural ingredients that can replace the synthetic components of cosmetics. The advantage of these compounds is that they exhibit antioxidant, anti-inflammatory, and soothing properties, enhancing the product’s value in addition to their antimicrobial properties.

Recently, essential oils (EOs) have gained increasing attention due to their therapeutic and preservative properties, which are beneficial for health and are generally considered safe according to the United States Agency for Food and Drug Products (Fancello et al., 2016). The EOs are natural substances that are composed of volatile secondary metabolites (Salehi et al., 2018). The study of the EOs’ chemical composition and the evaluation of their biological activities are necessary to confirm their use as preservatives in the food, pharmaceutical, and cosmetic fields. The genus Thymus is considered to be one of the eight most important genera in the Lamiaceae family, comprising approximately 215 species that are native to the Mediterranean basin (Pirbalouti et al., 2015). The majority of these oils are characterized by their richness in oxygenated monoterpenes, in particular, phenolic compounds such as thymol and its isomer carvacrol, accompanied by other more or less biologically active compounds such as eugenol, p-cymene, terpinene, linalool, geraniol, and borneol (Nikolić et al., 2014). Several studies linked Thymus EO’s chemical composition to its antimicrobial and antioxidant activities (Ballester-Costa et al., 2017).

*Thymus broussonetii*, endemic to Morocco, is an erect shrub with erect stems. Its leaves are stem-like, broadly ovate-lanceolate, and punctuated on both sides. Its flowers are wide and often purple-colored, and it is characterized by dense male inflorescences (Benabid, 2000). The severe exploitation to which this species is exposed can lead to its rarefaction and/or disappearance (Mehdioui and Kahouadji, 2007; Ozudogru et al., 2011). There is no study on the variation in the chemical composition of *T. broussonetii* EO. The Mediterranean flora, in general, and Morocco, in particular, boast a diversified flora with an endemism rate equal to 878 species, including several species of plants that have been little or not studied at all (Fennane and Tattu, 2008; Rankou et al., 2013). The present study is a contribution to the chemical composition and the antioxidant, antimicrobial, and preservative efficacy of *T. broussonetii* essential oil (EO) in topical cream formulations using the challenge test proposed by the European Pharmacopoeia against selected standard microorganisms that represent the potential contaminants of cosmetic products during manufacture or use. To the best of our knowledge, this is the first work to evaluate the preservative efficacy of the Moroccan endemic plant *T. broussonetii* Boiss.

## 2 Results and discussions

### 2.1 Chemical analysis

**FIGURE 1:**
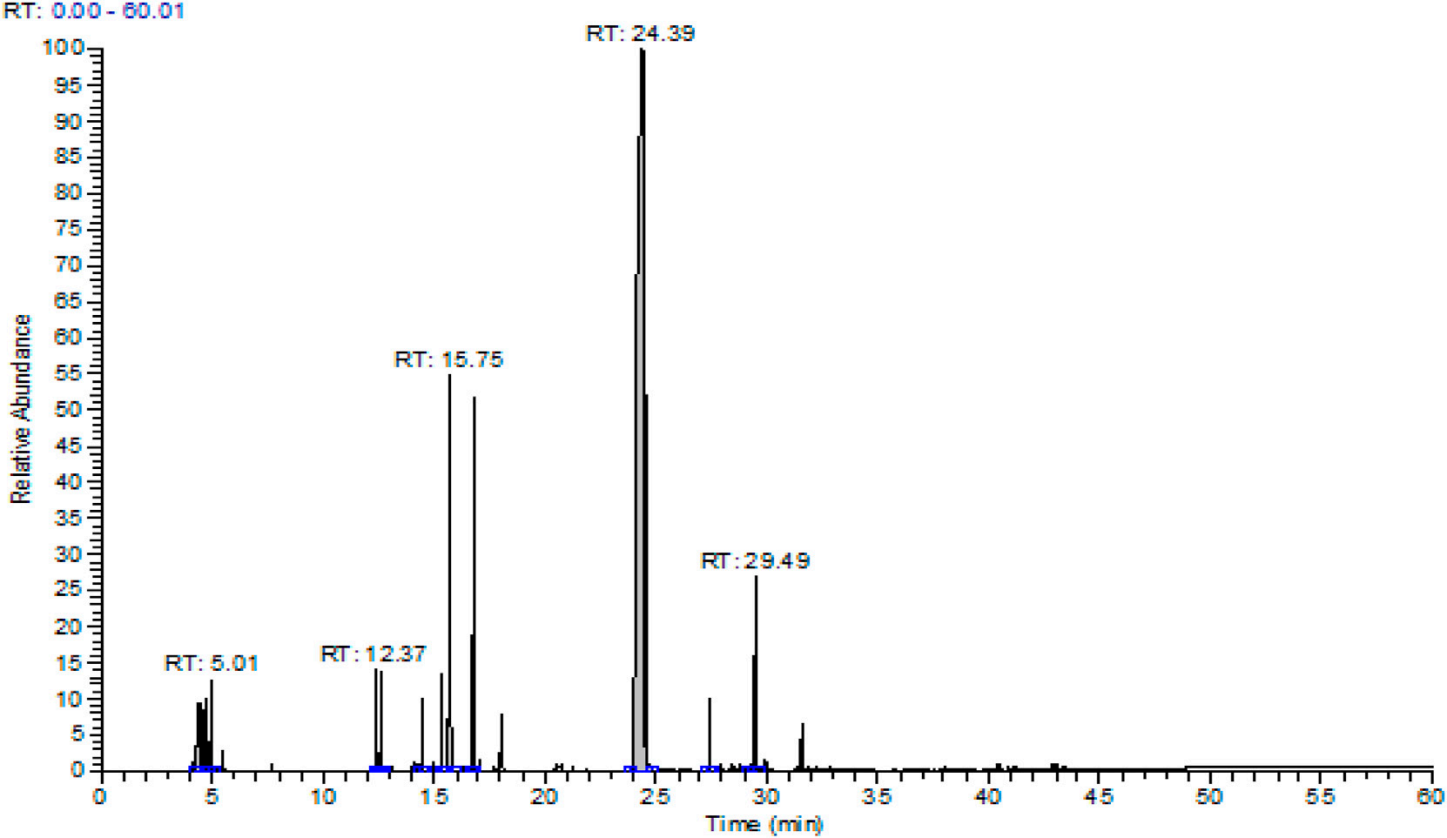
Chromatogram of *Thymus broussonetii* essential oil.

Essential oil obtained from the aerial part of *T. broussonetii* yielded 2.05% (v/w). The gas chromatography/mass spectrometry (GC/MS) analysis resulted in the identification of 11 compounds, representing more than 99.78% of the total oils and falling into the following three classes: monoterpene hydrocarbons, oxygenated monoterpenes, and sesquiterpene hydrocarbons (Table 1). Oxygenated monoterpenes such as thymol (63.09%) were the dominant constituents, followed by the two monoterpene hydrocarbons *p*-cymene (11%) and γ-terpinene (8.99%) Figure 1. The results showed that thymol was the most abundant compound in thyme oil. In contrast, p-cymene and γ-terpinene are two precursors of thymol (Nouri and Esmaeilian, 2012).

**TABLE 1:** Chemical composition (%) of *Thymus broussonetii* essential oil.

Peak	Major constituents	^a^RT (min)	Percentage (%)
1	5-Oxotetrahydrofuran-2-carboxylic acid	4.70	6.90
2	α-Thujene	5.01	1.80
3	α-Phellandrene	12.37	1.10
4	α-Pinene	12.63	1.08
5	3-Carene	14.46	0.88
6	Terpinen-4-ol	15.35	1.21
7	p-Cymene	15.75	11.0
8	γ-Terpinene	16.8	8.99
9	Thymol	24.39	63.09
10	(*E*)-Caryophyllene	27.43	0.88
11	Aromadendrene	29.49	2.85
Total			99.78

^a^ RT, Retention time.

### 2.2 Antioxidant activity

The essential oil of *T. broussonetii* obtained from *in vitro* cultivated plants (Nordine et al., 2013) was subjected to screening for its possible antioxidant activity by three methods, namely, DPPH free radical scavenging, ferric ion reduction, and β-carotene–linoleic acid assays.

The results are illustrated in Table 2. EOs are qualified as natural antioxidants because of their ability to reduce and/or prevent the formation of free radicals. According to the IC_50_ values presented in Table 2, the antioxidant power of quercetin and butylated hydroxytoluene (BHT) is greater than that of the studied thyme. The tested *T. broussonetii* essential oil exhibited significant antioxidant activity, as indicated by the minimum inhibitory concentration (IC_50_). In addition, tests show that the EO has a good affinity with Fe3+ ions in the FRAP method. The antioxidant activity of *T. broussonetii* EO has already been indicated in several works, including Radi et al. (2021), Hamdani et al. (2014), El Bouzidi et al. (2013), and Jamali et al. (2012).

**TABLE 2:** IC_50_ of *T. broussonetii* EO, quercetin, and of BHT standard measured by DPPH, FRAP, and β-carotene methods. Data are expressed as the mean ± SD. The experiment was performed in a minimum of three replicates.

	IC_50_ (µg/mL)		
	DPPH	FRAP	β-carotene
*T. broussonetii*	210 ± 0.3	84 ± 0.21	145 ± 0.1
BHT	4.21 ± 0.08	7.09 ± 0.1	4.3 ± 0.33
Quercetin	1.07 ± 0.01	2.29 ± 0.1	0.95 ± 0.33

Furthermore, the results suggest that essential oils from *T. broussonetii* could be used as natural antioxidants in cosmetics and food systems. Generally, the antioxidant activity is the result of the interaction between all the chemical components of the EO (alcohols, phenols, and terpene and ketone compounds) acting in an antagonistic or synergistic way. Many studies have established an important relationship between the chemical composition of essential oils and their antioxidant activity, and it has been reported that the antioxidant activity of essential oils is related to their chemical compositions, particularly the presence of compounds containing a hydroxyl function as well as terpene alcohols and phenolic compounds (Xiaohua et al., 2023; Bouhdid et al., 2008; Fayed, 2009; Zhuang et al., 2009; Miladi et al., 2013).

According to this study, it is evident that the high antioxidant capacity of the studied EO of *T. broussonetii* is linked to its content of thymol, ɣ-terpinene, p-cymene, and α-pinene. In addition, the presence of minority compounds such as aromatic alcohols, ethers, ketones, and esters in the studied EO can influence the antioxidant activity.

### 2.3 Antimicrobial activity

**FIGURE 2:**
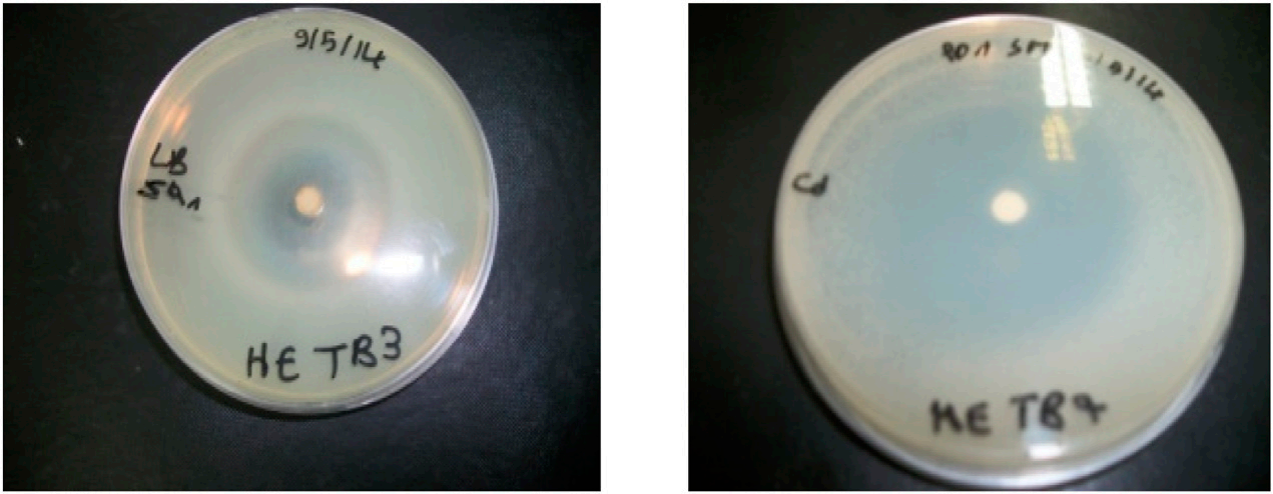
Plate inhibition zone assay showing the antimicrobial activity of *Thymus broussonetii* essential oil against *S. aureus* and yeast.

As shown in Tables 3 and 4, the antimicrobial assays indicated that the essential oil was more effective against Gram-positive bacteria than against Gram-negative bacteria; the MICs of the essential oil against bacteria and fungi were 0.02%–1% (Figure 2). However, the essential oil exhibited a significant antibacterial effect against *Staphylococcus aureus* and *Escherichia coli*. Furthermore, GC/MS analysis showed that the major compound of the essential oil was thymol, which is proven to be responsible for the antimicrobial properties (Nielsen and Rios, 2000; Kiran Babu et al., 2007).

**TABLE 3:** Antimicrobial activity of *T. broussonetii* essential oil.

	Inhibition zone diameter (mm)^a^				
	Bacterial strains			Fungal strains	
	*S. aureus*	*E. coli*	*P. aeruginosa*	*C. albicans*	*A. brasiliensis*
*Thymus broussonetii*	48.00 ± 1.15^***^	28.00 ± 1.15^**^	12.00 ± 1.15	42.33 ± 1.45	NZ
Streptomycin	12.67 ± 0.3	15.33 ± 0.3	21.66 ± 3.8	-	-

^a^ Diameter of the inhibition zone including disc diameter of 6 mm by the agar disk diffusion method at a concentration of 10 µL of oil/disc and streptomycin 10 µg/disc; NZ, no measurable zone of inhibition; -, not tested,*, the mean difference is significant at the 0.05 level.

**TABLE 4:** Antimicrobial activity of *T. broussonetii* essential oil using MIC and MBC methods.

Strains	Essential oil (%)		Antibiotic (µg/mL)	
	*T. broussonetii*		Streptomycin	
	MIC	MMC	MIC	MMC
*S. aureus*	0.02	0.03	4.00	4.00
*E. coli*	0.03	>1	8.00	64.0
*P. aeruginosa*	1.00	8.00	32.0	32.0
*C. albicans*	0.02	0.03	-	-
*A. brasiliensis*	0.02	0.06	-	-

MIC, minimum inhibitory concentration; MMC, minimum microbiocidal concentration; negative control, bacterial suspensions and Mueller–Hinton broth for bacteria or Sabouraud dextrose broth for fungi supplemented with agar (0.15% w/v); -, not tested.

The phenolic compound thymol, which is present in high concentration (63.09%) in our essential oil sample, is well-known for its broad-spectrum antimicrobial activity (Chami et al., 2005; Mothana et al., 2011; Castilho et al., 2012; Kurdelas et al., 2012). Moreover, previous studies have also reported the potent antimicrobial efficacy of thymol (Nostro et al., 2004; Gutierrez-Larrainzar et al., 2012). The presence in the hydroxyl group of thymol and a system of delocalized electrons in its chemical structure is important for its antimicrobial effect against Gram-negative or positive bacteria (Nazzaro et al., 2013; Saad et al., 2013).

The mode of action of thymol, a phenolic monoterpenoid, has received a lot of attention from researchers. However, thymol interacts with the cell membrane, affecting its permeability and leading to the loss of membrane potential, cellular uptake of ethidium bromide, and leakage of potassium ions, ATP, and carboxyfluorescein (Xu et al., 2008). Other constituents have also been reported for their antimicrobial activity, such as (*E*)-caryophyllene (Maggi et al., 2009; Randrianarivelo et al., 2009; El Bouzidi et al., 2013). Other reports showed that terpinen-4-ol has a higher antibacterial effect than *p*-cymene (Kasujja, 2021).

### 2.4 Stability study

**FIGURE 3:**
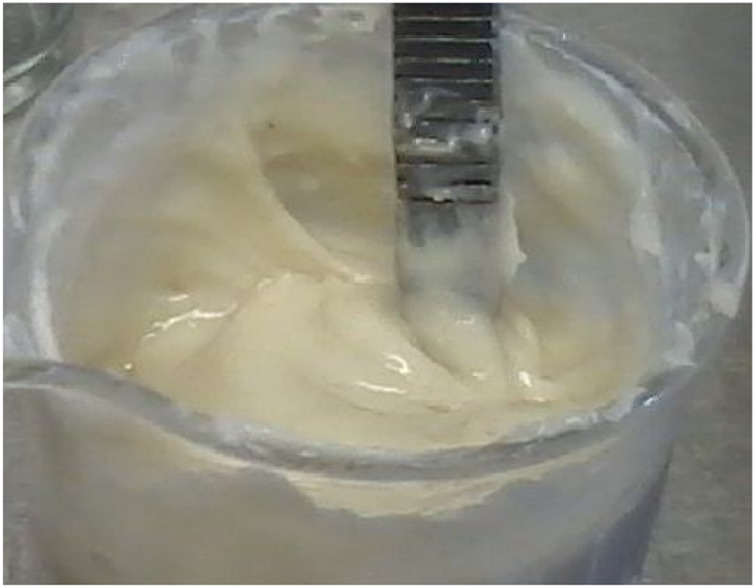
Visual appearance of the formulation of essential oil of *T. broussonetii.*

The visual appearance of the formulation was checked at the time of preparation and after 1 month. No significant difference in visual appearance was found after 1 month from the time of preparation (Figure 3). The color of the cream formulation at the time of preparation and after 1 month of storage at 25°C, 37°C, and 4°C was observed to be “white.” The pH evaluation is important to check the stability of a cream formulation. The pH values were not different at all temperatures for a period of 28 days. The pH values after this period were 5.7 at 25°C and 4°C and 5.8 at 37°C.

### 2.5 Challenge test

**FIGURE 4:**
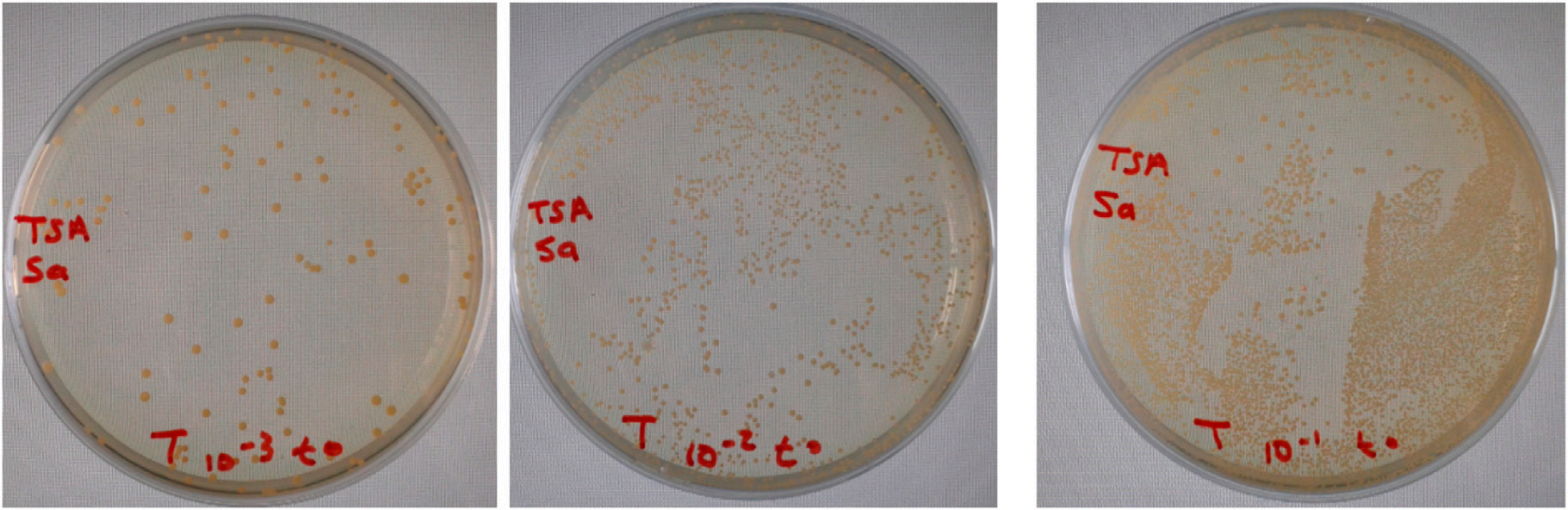
Example of bacterial challenge testing against *S. aureus* in the cream formulation.

**FIGURE 5:**
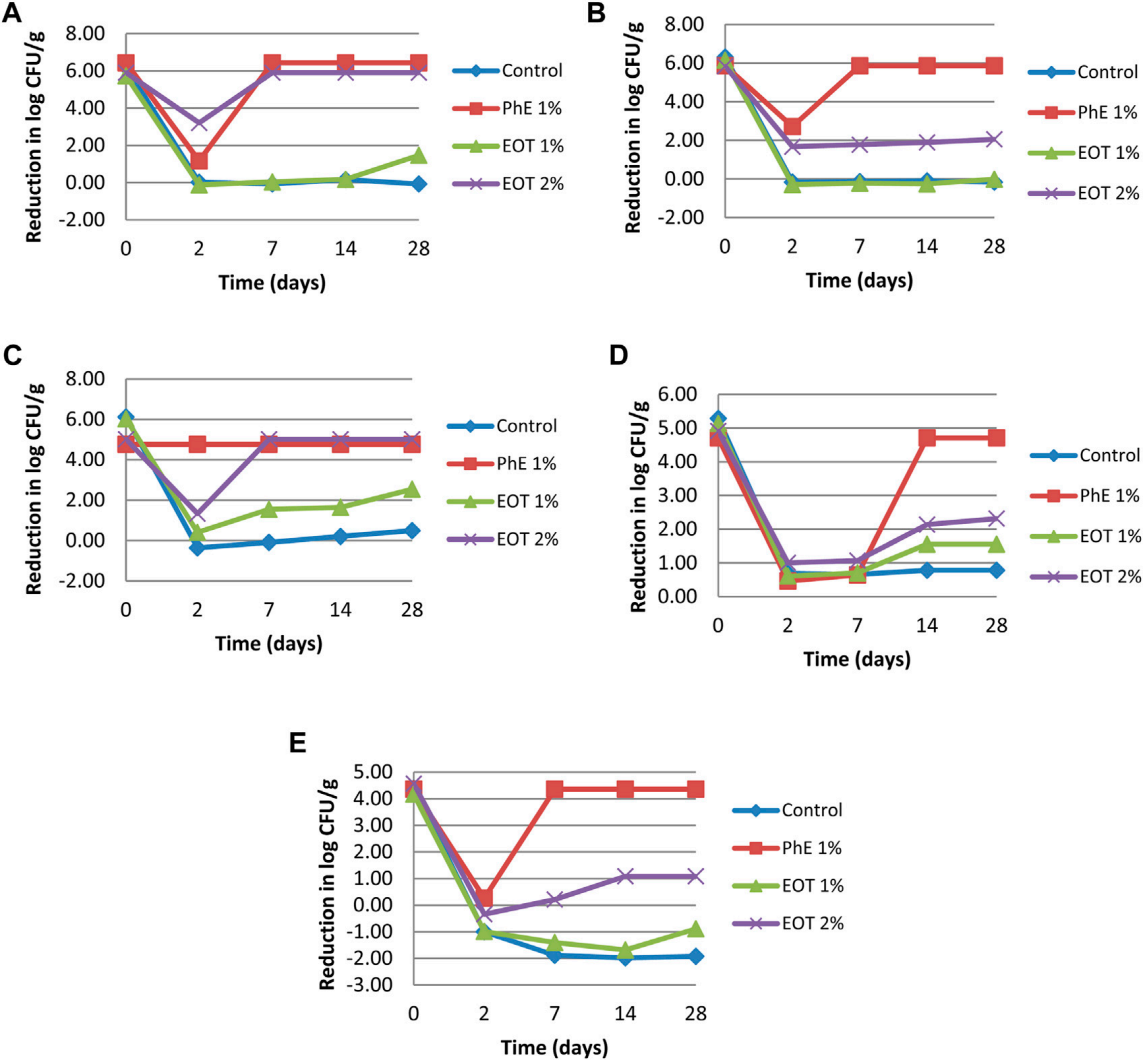
Reduction in log measured during the challenge test for each of the bacterial and fungal strains. **(A)** *S. aureus*, **(B)** *P. aeruginosa*, **(C)** *E. coli*, **(D)** *A. brasiliensis*, and **(E)** *C. albicans;* EOT 1%, cream with 1% essential oil, EOT 2%, cream with 2% (v/w) essential oil, PhE 1%, cream preserved with 1% (v/w) phenoxyethanol; control, cream without any preservative.

Preservatives are ingredients used to limit microbial growth and survival in cosmetic products. However, some chemical preservatives present adverse effects, such as hypersensitivity. Chemical preservatives have been replaced by other antimicrobial agents, such as essential oils and extracts (Gutierrez-Larrainzar et al., 2012). Based on the test results of the minimum inhibitory concentration and considering that high concentrations of preservatives can cause some adverse effects, the cream used in the challenge was added to 1% and 2% of *T. broussonetii* essential oil. The present study indicated a rapid decline in the number of CFU g^-1^ during the first few times evaluated after the contamination of the cream by all tested microbes (Figure 4, 5).

Our results demonstrated that the essential oil of *T. broussonetii* evaluated at 2% (v/w) in a cream formulation succeeded in satisfying the A criteria for preservation efficacy against all microbial strains (*S. aureus, E. coli*, and *Aspergillus brasiliensis*) except *Pseudomonas aeruginosa* (1.78 log reduction in the number of CFU g^-1^ after the 7th day of evaluation) and *Candida albicans* (1.09 log reduction in the number of CFU g^-1^ after 14 days of evaluation) in the cream study compared to phenoxyethanol 1% (v/w). Compared to other species of thyme, Manou et al. (1998) reported that the challenge test of *Thymus vulgaris* essential oil at 3% (v/w) revealed an unsatisfactory effect of preservation, and the required criteria were satisfactory only against the bacterial strains and partially against yeast but not against *Aspergillus niger*.

In general, in bacteria, structural changes in the outer membrane protein (OMP) composition may have an effect on the adhesive ability and pathogenic properties of the organisms. *P. aeruginosa* can develop an intrinsic resistance to a wide range of biocides, which is associated with the nature of its outer membrane (Verdial et al., 2023). This strain is known to be very persistent and predominant in cases of drug and cosmetic spoilage. In this context, we can explain the unsatisfactory effect of the essential oil of *T. broussonetii* against *P. aeruginosa.*

### 2.6 Molecular docking study

In the study of antioxidant activity, the molecule that demonstrated the highest effectiveness against NADPH was thymol, with a glide score of −6.388 kcal/mol. In the analysis of antimicrobial activity, the most active molecule against *S. aureus* nucleoside diphosphate kinase was terpinen-4-ol, with a score of −6.008 kcal/mol. Thymol also exhibited significant activity against *E. coli* beta-ketoacyl-[acyl-carrier protein] synthase, with a score of −6.83 kcal/mol. Additionally, in the case of the *P. aeruginosa* LasR ligand-binding domain, thymol was identified as the most active molecule, with a glide score of −7.406 kcal/mol. Lastly, the most active molecule against *C. albicans* was aromadendrene, with a glide score of −7.084 kcal/mol (Table 5).

**TABLE 5:** Docking results with ligands in different receptors.

		Glide G score (Kcal/mol)				
		Antioxidant activity	Antimicrobial activity			
		2CDU	3Q8U	1FJ4	2UV0	5FSA
Essential oil compound	(E)-Caryophyllene	−3.589	−4.157	−5.064	−5.934	−7.025
	3-Carene	−4.245	−4.57	−5.852	−6.909	−5.526
	5-Oxotetrahydrofuran-2-carboxylic acid	−5.579	−5.549	−6.432	−5.832	−5.783
	α-Phellandrene	−4.854	−5.607	−6.114	−6.776	−5.805
	α-Pinene	−3.789	−4.136	−5.663	−6.494	−4.959
	α-Thujene	−4.762	−4.967	−6.189	−6.691	−5.595
	Aromadendrene	−4.688	−5.57	−6.265	−7.173	−7.084
	γ-Terpinene	−4.938	−5.155	−5.905	−6.871	−5.094
	p-Cymene	−5.085	−5.152	−5.794	−6.923	−5.904
	Terpinen-4-ol	−4.667	−6.008	−5.862	−6.839	−6.14
	Thymol	−6.388	−5.547	−6.83	−7.406	−6.526

**FIGURE 6:**
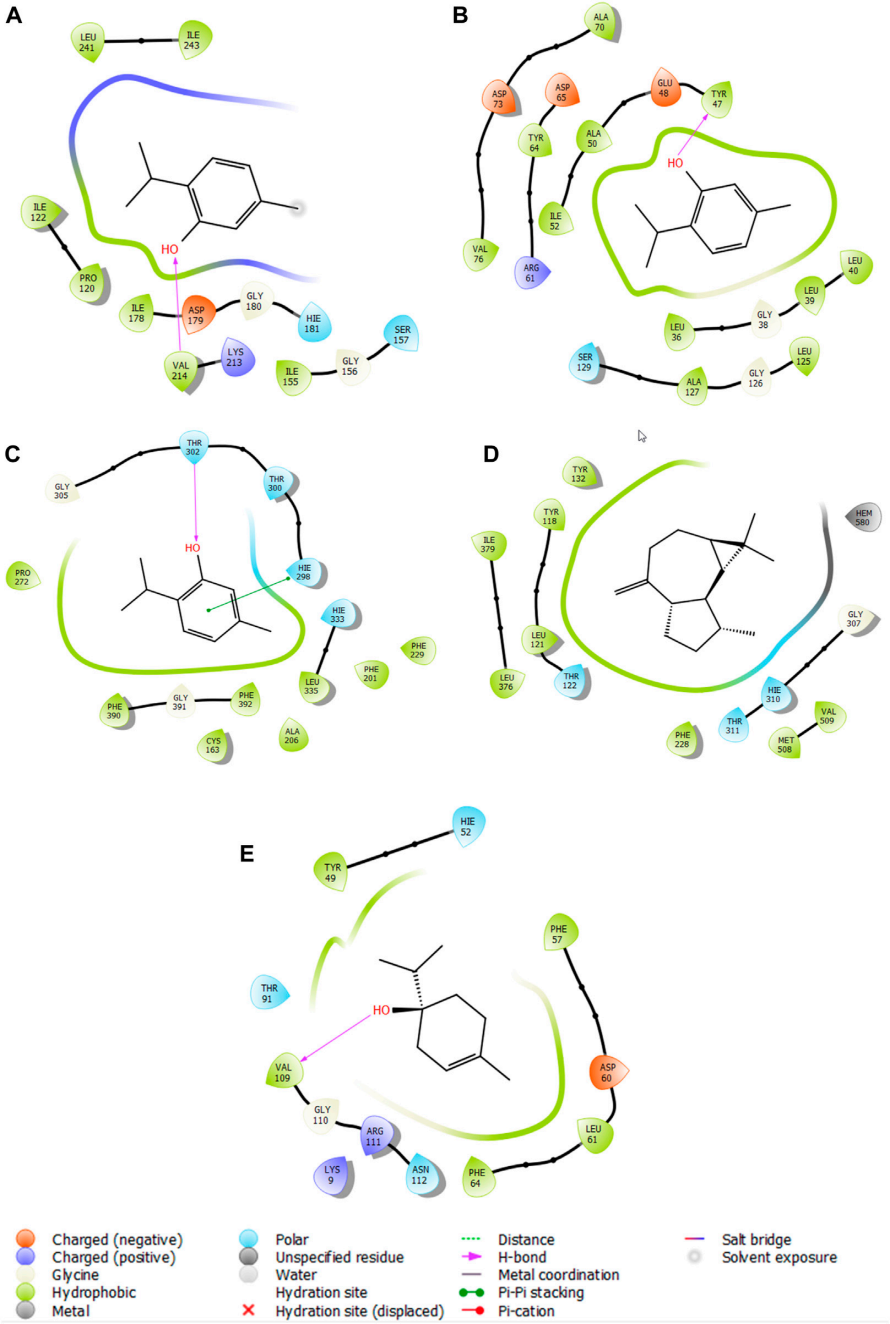
2D viewer of ligand interactions with the active site. **(A–C)** Thymol interactions with the active site of NADPH, *P. aeruginosa* LasR ligand-binding domain, and *E. coli* beta-ketoacyl-[acyl-carrier protein] synthase, respectively. **(D)** Aromadendrene interactions with the active site of *C. albicans*. **(E)** Terpinen-4-ol interactions with the active site of *S. aureus* nucleoside diphosphate kinase.

Thymol is the most active molecule responsible for antioxidant and antimicrobial activities. In the active site of NADPH, thymol established a single hydrogen bond with the residue VAL 214, while it established two hydrogen bonds in the active site of glycogen synthase kinase 3 with the residue VAL 135. Moreover, in the active site of *E. coli* beta-ketoacyl-[acyl-carrier protein] synthase, it established a single hydrogen bond with the residue THR 302 and a Pi–Pi stacking bond with the residue HIE 298 (Figure 6).

5-Oxotetrahydrofuran-2-carboxylic acid was the most active molecule in the active site of human casein kinase 1 alpha 1, and it established two hydrogen bonds with residues ARG 186 and LYS 225 and a salt bridge type bond with residue LYS 225. Meanwhile, terpinen-4-ol was the most dynamic molecule in the active site of *S. aureus* nucleoside diphosphate kinase, and it established a single hydrogen bond with the residue VAL 109 (Figure 6).

### 2.7 ADME/toxicity prediction

The bioavailability of an active compound is determined by its absorption, distribution, metabolism, and excretion (ADME), and it is influenced by the physicochemical properties of the compound. All compounds identified in the *T. broussonetii* EO were evaluated for their ADME properties, with all compounds having a molecular mass below 500 mol. The number of donors and acceptors of hydrogen bonds for the essential oil were within the acceptable limits (≤5 and ≤10, respectively) (Table 6). The total surface area accessible to the solvent affects the oral bioavailability, and all molecules had acceptable values ranging from 300 to 1,000. The blood–brain partition coefficient determines a molecule’s ability to cross the blood–brain barrier, with an acceptable range for the predicted coefficient being −3 to 1.2. All the selected molecules met this criterion. The predicted percentage of oral absorption for all the compounds was approximately 100%, as indicated in Table 6.

**TABLE 6:** ADME prediction of the compounds identified in the *T. broussonetii* essential oil.

	mol MW	SASA	Donor HB	accptHB	QPlogPw	QPlogPo/w	QPlogS	CIQPlogS	QPPCaco	QPlogBB	QPlog Kp	Percent human oral absorption
Aromadendrene	204.355	455.603	0	0	−0.151	5.134	−6.141	−6.141	9906.038	1.043	−1.412	100
(E)-Caryophyllene	204.355	449.878	0	0	−0.363	5.037	−6.131	−6.131	9906.038	1.033	−1.414	100
Thymol	150.22	391.249	1	0.75	3.393	3.295	−2.321	−2.191	3835.969	0.088	−1.779	100
γ-Terpinene	136.236	390.401	0	0	−0.2	4.045	−4.128	−4.316	9906.038	0.863	−1.229	100
p-Cymene	134.221	383.117	0	0	0.46	3.648	−3.689	−4.128	9906.038	0.7	−0.969	100
Terpinen-4-ol	154.252	386.76	1	0.75	2.803	2.95	−2.53	−2.014	5946.679	0.262	−1.638	100
3-Carene	136.236	366.086	0	0	−0.49	3.614	−3.988	−3.988	9906.038	0.864	−1.413	100
α-Pinene	136.236	366.057	0	0	−0.499	3.613	−3.986	−3.986	9906.038	0.867	−1.418	100
α-Phellandrene	136.236	381.942	0	0	−0.055	3.908	−4.13	−4.207	9906.038	0.771	−1.097	100
5-Oxotetrahydrofuran-2-carboxylic acid	130.1	283.245	1	5	7.678	−0.457	−0.167	−0.359	71.418	−0.641	−4.428	57.45
α-Thujene	136.236	378.814	0	0	−0.638	3.836	−4.187	−4.187	9906.038	0.874	−1.289	100

^a^ Mass of molecules (acceptable range: 500 mol).

^b^ Donor of hydrogen bonds (acceptable range: ≤5).

^c^ Acceptor of hydrogen bonds (acceptable range: ≤10).

^d^ Total solvent accessible surface area using a probe with a 1.4 radius (acceptable range: 300–1000 radius).

^e^ Predicted octanol/water partition coefficient (acceptable range: −2–6.5).

^f^ Predicted blood–brain partition coefficient (acceptable range: −3–1.2).

^g^ Predicted aqueous solubility, S in mol/dm−3 (acceptable range: −6.5–0.5).

^h^ Predicted human oral absorption on 0%–100% scale (<25% is poor and >80% is high).

Toxicity studies are important for evaluating the potential harm that a substance or product can cause to living organisms, including humans, animals, and the environment. These studies are critical for ensuring the safety of drugs, chemicals, pesticides, food additives, and consumer products.

In our study, the prediction of the compounds identified in *T. broussonetii* EO showed potential immunotoxicity for (E)-caryophyllene, mitochondrial membrane potential toxicity for thymol, and carcinogenicity for p-cymene (Table 7).

**TABLE 7:** Toxicity prediction for all compounds identified in *T. broussonetii* essential oil*.*

Target	Aromadendrene	(E)-Caryophyllene	Thymol	γ-Terpinene	p-Cymene	Terpinen-4-ol	3-Carene	α-Pinene
Hepatotoxicity	Inactive	Inactive	Inactive	Inactive	Inactive	Inactive	Inactive	Inactive
Carcinogenicity	Inactive	Inactive	Inactive	Inactive	**Active**	Inactive	Inactive	Inactive
Immunotoxicity	Inactive	**Active**	Inactive	Inactive	Inactive	Inactive	Inactive	Inactive
Mutagenicity	Inactive	Inactive	Inactive	Inactive	Inactive	Inactive	Inactive	Inactive
Cytotoxicity	Inactive	Inactive	Inactive	Inactive	Inactive	Inactive	Inactive	Inactive
AhR	Inactive	Inactive	Inactive	Inactive	Inactive	Inactive	Inactive	Inactive
AR	Inactive	Inactive	Inactive	Inactive	Inactive	Inactive	Inactive	Inactive
AR-LBD	Inactive	Inactive	Inactive	Inactive	Inactive	Inactive	Inactive	Inactive
Aromatase	Inactive	Inactive	Inactive	Inactive	Inactive	Inactive	Inactive	Inactive
ER	Inactive	Inactive	Inactive	Inactive	Inactive	Inactive	Inactive	Inactive
ER-LBD	Inactive	Inactive	Inactive	Inactive	Inactive	Inactive	Inactive	Inactive
PPAR-Gamma	Inactive	Inactive	Inactive	Inactive	Inactive	Inactive	Inactive	Inactive
nrf2/ARE	Inactive	Inactive	Inactive	Inactive	Inactive	Inactive	Inactive	Inactive
HSE	Inactive	Inactive	Inactive	Inactive	Inactive	Inactive	Inactive	Inactive
MMP	Inactive	Inactive	**Active**	Inactive	Inactive	Inactive	Inactive	Inactive
P53	Inactive	Inactive	Inactive	Inactive	Inactive	Inactive	Inactive	Inactive
ATAD5	Inactive	Inactive	Inactive	Inactive	Inactive	Inactive	Inactive	Inactive

AhR, aryl hydrocarbon receptor; AR, androgen receptor; AR-LBD, androgen receptor ligand-binding domain; ER, estrogen receptor alpha; ER-LBD, estrogen receptor ligand-binding domain; PPAR-gamma, peroxisome proliferator activated receptor gamma; nrf2/ARE, nuclear factor (erythroid-derived 2)-like 2/antioxidant responsive element; HSE, heat shock factor response element; MMP, mitochondrial membrane potential; p53, phosphoprotein (tumor suppressor); ATAD5, ATPase, family AAA domain-containing protein.

ProTox-II is a tool used for predicting the toxicity of chemical compounds. It is based on a set of predictive models that use various chemical and molecular descriptors to estimate the potential toxicity of a given compound. These models were developed using machine learning techniques and are trained on large datasets of known toxic and non-toxic compounds. The models used in ProTox-II are designed to predict various types of toxicity, including acute toxicity, skin and eye irritation, and mutagenicity. Tox21 (toxicology in the 21st century), used by ProTox-II, is a research program that aims to develop better ways to predict the potential toxicity of nuclear receptor signaling pathways and stress response pathways.

## 3 Conclusion

Several types of EOs and their individual components are used as natural antimicrobial compounds in order to reduce the impact of microbial activities in cosmetic products. Thyme essential oil has antimicrobial, antifungal, and antioxidant properties. Its antimicrobial and antioxidant activity has been studied almost exclusively by chemical testing in order to be able to use it for cosmetic preservation purposes. Compared to the Gram-negative bacteria, the Gram-positive bacterial strains are more sensitive to the EO. Thymol, p-cymene, and γ-terpinene are the major components present in the EO, which are responsible for maximizing the antimicrobial activity. According to the IC_50_ values, the tested *T. broussonetii* essential oil exhibited important antioxidant activity.

Our results indicate that *T*. *broussonetii* essential oil evaluated at 2% (v/w) in a cream formulation succeeded in satisfying the A criteria for preservation efficacy against *S. aureus*, *E. coli*, and *A. brasiliensis* but exhibited less efficacy against *P. aeruginosa* and *C. albicans* compared to the synthetic preservative phenoxyethanol at 1% (v/w). The molecular docking study revealed that *T. broussonetii* compounds might exert an antioxidant effect through NADPH oxidase inhibition. *In silico* results demonstrated that the antimicrobial activity of T. broussonetii EO is mostly attributed to thymol, terpinen-4-ol, and aromadendrene. These results indicate that the essential oil of *T. broussonetii* possesses important antimicrobial and antioxidant properties and can, therefore, be used as a natural preservative ingredient in the cosmetic industry.

## 4 Materials and methods

### 4.1 Plant material

The aerial parts of *T. broussonetii* Boiss. (Lamiaceae) obtained from an *in vitro* cultivated plant (Nordine et al., 2014) were freshly harvested and collected on April 2023 in the experimental garden of the National Agency of Medicinal and Aromatic Plants. This plant was produced through *in vitro* clonal propagation through direct shoot organogenesis. The plant was identified by a botanist, and a voucher specimen (INP. 564) was deposited at the herbarium of the National Institute of Medicinal and Aromatic Plants (NIMAP), Taounate, Morocco.

### 4.2 Essential oil extraction

The EO from the aerial part of *T. broussonetii* was extracted by hydrodistillation using a Clevenger-type apparatus (VWR, Radnor, PA, United States) for 3 hours. The process was repeated three times for each 100 g of the plant sample. The obtained essential oil was dried over anhydrous sodium sulfate. It was then stored at a temperature of 4°C in the dark until it was used.

### 4.3 Gas chromatography/mass spectrometry analysis of the essential oil

GC/MS analysis was performed using a gas chromatograph (TRACE GC Ultra) (Conquer Scientific, Poway, CA, United States) fitted to a mass spectrometer (Polaris Quadrupole-Ion Trap MS) (Conquer Scientific, Poway, CA, United States) using a Varian capillary column (TR5 CP-Sil 5 CB; 50 m length, 0.32 mm diameter, and film thickness 1.25 µm). Fragmentation was performed by electron impact at 70 eV. Helium (1.5 mL/min) was used as the carrier gas. A split-type injector was heated to a temperature of 200°C. The volume injected was 1 μL. The column temperature was programmed from 40°C to 280°C for 5 °C/min. The components were identified by comparison of their mass spectra with authentic reference compounds (1 mg/mL) where possible, by reference to WILEY275, NIST-MS, and the Adams terpene library (Adams, 2001), and by comparison of their retention time (RT) with those of authentic compounds or literature data. For semi-quantification purposes, the normalized peak area of each compound was used without any correction factors to establish abundances.

### 4.4 Formulation of the cream

In an attempt to determine the preservative effect exerted by the essential oil, the challenge test was performed in the following four final formulations: cream preserved with 1% essential oil (EOT 1%), cream preserved with 2% essential oil (EOT 2%), cream preserved with 1% synthetic preservative phenoxyethanol (PhE), and the same cream without any preservative as the control group. The composition and pH of the cream are indicated in Table 8.

**TABLE 8:** Composition of the cream used in the challenge test.

	Cream formula	Composition (%)
Oily phase	Beeswax	22.0
	Almond oil	49.2
	Polysorbate 80	0.80
Aqueous phase	Water	q.s 100
pH of the cream	5.6	

### 4.5 Stability study

#### 4.5.1 Visual appearance

The visual appearance (color and texture) of the cream was determined by taking 5 g of cream from each set (stored at 25°C, 37°C, and 4°C) in three transparent glass jars and checking the visual appearance. This process was carried out at the time of cream preparation (at zero time) and thereafter for 1 month.

#### 4.5.2 pH of the formulation

The pH of the formulation was determined using the pH meter (Mettler Toledo Ingold Inc., Billerica, MA) (Bates Roger, 1973). The pH was checked at the time of cream preparation (zero time) and thereafter for 1 month. The cream jars stored at 25°C, 37°C, and 4°C were selected to check the pH of the cream to ensure its stability at different temperatures. Measurements were taken in triplicate. The pH meter was calibrated with standard buffer solutions (pH 4, 7, and 10) before each use.

### 4.6 Antioxidant activity

#### 4.6.1 DPPH free radical scavenging activity

The DPPH radical-scavenging assay was determined according to the method reported by Burits and Bucar (2000). Essential oil (50 µL) at different concentrations (26.96–863 mg/mL) or MeOH (control) was mixed with 2 mL of 2,2-diphenyl-1-picrylhydrazyl (DPPH) methanol solution (60 mg/L). After 20 min of incubation in darkness at ambient temperature, the absorbance was measured at 517 nm. Butylated hydroxytoluene (BHT) and quercetin were used as the positive controls, all analyses were carried out in triplicate, and the results were expressed as the mean ± SD. The percentage inhibition of the DPPH radical was calculated according to the following formula:

$$\%\text{ Inhibition}=\left(A_{b}\;–\;A_{a}\;/A_{b}\right)\times 100.$$

Here, A_b_ is the absorption of the control sample (DPPH + methanol) and A_a_ is the absorption of the tested oil. The sample concentration providing 50% inhibition (IC_50_) was calculated by plotting the inhibition percentages against the concentrations of the sample.

#### 4.6.2 Reducing power determination

The reducing power of the essential oil was determined according to the method reported by Miraliakbari and Shahidi (2008). Different concentrations of the oil in methanol (29.37–940 mg/mL) were mixed with phosphate buffer (500 μL, 0.2 mol/L, pH 6.6) and potassium ferricyanide [K_3_Fe(CN)_6_] (500 μL, 1%). The mixture was incubated at 50°C for 20 min. After incubation, 500 µL of trichloroacetic acid (10%, w/v) was added to the mixture to stop the reaction. The mixture was centrifuged at 650 *g* for 10 min, and then 500 µL of the supernatant was mixed with distilled water (500 µL) and ferric chloride (100 μL, 1 mg/mL). The oil concentration providing 0.5 absorbance (IC_50_) was calculated by plotting the absorbance at 700 nm against the corresponding oil concentration. BHT and quercetin were used as the reference compounds. The test was carried out in triplicate, and the IC_50_ values were reported as the means ± SD.

#### 4.6.3 β-Carotene–linoleic acid assay

In this assay, the antioxidant capacity was determined by measuring the inhibition of the volatile organic compounds and the conjugated diene hydroperoxides arising from linoleic acid oxidation (Sahin et al., 2004). A stock solution of the β-carotene–linoleic acid mixture was prepared as follows: 0.5 mg of b-carotene was dissolved in 1 mL of chloroform (HPLC grade), and 25 µL of linoleic acid and 200 µL of Tween 40 were added. Chloroform was completely evaporated using a vacuum evaporator. Then, 100 mL of distilled water saturated with oxygen (30 min, 100 mL min^-1^) was added with vigorous shaking; 2.5 mL of this reaction mixture was dispersed into test tubes, and 350 µL portions of the essential oil prepared in methanol at different concentrations (19–302.7 μg/mL) were added. The emulsion system was incubated for up to 2 h at 50°C. The same procedure was repeated with the positive control BHT, quercetin, and a blank. After this incubation period, the absorbance of the mixtures was measured at 470 nm. Antioxidant capacities of the extracts were compared with those of BHT and quercetin at the same concentration, and the blank consisted of only 350 µL of methanol.

### 4.7 Antimicrobial activity

#### 4.7.1 Microbial strains

The standard microbial strains were procured from the American Type Culture Collection (ATCC). The Gram-positive bacteria *S. aureus* ATCC 29213, Gram-negative bacteria *E. coli* ATCC 25922 and *P. aeruginosa* ATCC 27853, yeast *C. albicans* ATCC 10231, and mold *A. brasiliensis* ATCC 16404 was used in the microbiological tests and as the challenged microorganisms in the preservative effectiveness test.

#### 4.7.2 Antimicrobial screening

The antimicrobial activity was performed using the agar-disk-diffusion method according to CLSI guidelines with some modifications (CLSI, 2012). Briefly, Petri dishes containing Mueller–Hinton agar (MHA) culture medium and Sabouraud dextrose agar (SDA) culture medium for bacteria and yeast, respectively, were inoculated with the microbial inoculums previously prepared. The disks (filter paper, 6 mm in diameter) placed in the center of each plate were impregnated with 10 μL of each essential oil. Negative control consisted of microbial suspensions and medium culture supplemented with agar (0.15% w/v). Streptomycin (10 µg/disc) was used as the positive control for the tested bacteria. The Petri dishes were placed at 4°C for 2 h to allow a better diffusion of molecules. After the plates were incubated at 37°C for 18–20 h for bacteria and at 30°C for 24–48 h for yeast, the diameters of the inhibition zones were measured (in mm), and the experiments were carried out in triplicate.

#### 4.7.3 Antifungal screening

The antifungal activity was evaluated by agar-well diffusion against *A. brasiliensis* with slight modification (Schlumbaum et al., 1986). An agar-plot or cylinder was cut aseptically with a sterile cork borer and deposited in the center of the agar surface plate for inoculation. Then, a hole with a diameter of 8 mm was punched aseptically with a sterile cork borer or a tip, and 80 µL of the essential oil solution was introduced into the well. Then, the agar plates were incubated, and the antifungal activity was evaluated by measuring the zone of inhibition of fungal growth surrounding the well in mm.

#### 4.7.4 Minimum inhibitory concentration

The determination of MIC was performed in a 96-well microplate using the micro-dilution assay according to Bouhdid et al. (2009), with slight modifications. First, each essential oil was serially diluted in Mueller–Hinton broth (MHB) for bacteria and Sabouraud dextrose broth (SDB) for fungi, supplemented with agar 0.15% (w/v), which was used as an emulsifier. The 12th well was considered the growth control. Then, 50 μL of bacterial inoculum was added to each well at a final concentration of 10^6^ CFU/mL. After incubation at 37°C for 18–20 h, 5 μL of resazurin was added to each well as the bacterial growth indicator. After further incubation at 37°C for 2 h, the bacterial growth was revealed by the change in coloration from purple to pink. The MIC value was determined as the lowest concentration that prevented a change in resazurin color. Assays were conducted in triplicate.

#### 4.7.5 Minimum microbiocidal concentration

The minimum microbiocidal concentration (MMC) (minimum bactericidal and fungicidal concentration) corresponded to the lowest concentration of the essential oil, yielding negative subcultures after incubation at 37°C for 24 h and 48 h to 5 days for yeast and mold, respectively. It is determined by spotting 2 μL from the negative wells on LB plates. Each assay was also conducted in triplicate.

### 4.8 Challenge test

The efficacy of the preservative test was determined following the standards proposed by the European Pharmacopoeia Commission concerning topical preparations, as described (European Pharmacopoeia Commission Efficacy of Antimicrobial Preservation, 2005). The cream formulations (20 g) were placed in sterile containers and separately inoculated with 0.2 mL of each bacterial and fungal suspension in order to obtain a final concentration of approximately 10^5^–10^6^ CFUg^−1^. The samples were gently shaken to ensure a homogeneous microorganism distribution and incubated at 25°C. Samples of 1 g were removed and placed into 9 mL of peptone solution (0.1%), and serial decimal dilutions were performed before inoculation in the microbial plates.

The same procedure was performed after 2, 7, 14, and 28 days of contact. Cell viability was determined using the plate count method in TSA or SDA, and the CFUs were counted after 2 or 5 days of incubation at 37°C and 25°C for bacteria and fungi, respectively. All determinations were performed in triplicate. The viability and growth ability of the inoculated cells were evaluated by a growth control, which consisted of 20 g cream samples without any preservatives separately inoculated with 0.2 mL of each bacterial and fungal suspension. In order to determine the preservative properties of the topical cream, a reduction of three log phases (3 log) from the initial bacterial count within 1 week and no increase thereafter up to 4 weeks is necessary. For fungi, a 2-log reduction within 2 weeks and no increase thereafter up to 1 month following the initial contamination is required.

### 4.9 Molecular docking

For the *in silico* evaluation of different biological activities of the *T. broussonetii* essential oil, we chose the enzyme NADPH for the antioxidant activity; human casein kinase 1 alpha 1 (CSNK1A1) and glycogen synthase kinase 3 for the healing activity; and *S. aureus* nucleoside diphosphate kinase, *E. coli* beta-ketoacyl-[acyl-carrier protein] synthase, *Pseudomonas aeruginosa* LasR ligand-binding domain, and sterol 14-alpha demethylase (CYP51) from *C. albicans* for the antimicrobial activity.

The molecular docking process was carried out using the Glide SP module of the Schrodinger Maestro program. This included using the program to execute LigPrep, PrepWizard, grid generation, and docking calculations and identifying key parameters such as the glide score, energy, emodel, and ligand efficiency.

The preparation of the ligands involved downloading the compounds identified in the *T. broussonetii* EO by GC/MS from PubChem in the SDF format. These ligands were then readied for docking calculations through the use of the LigPrep tool within the Maestro 11.5 version of the Schrödinger software program, utilizing the OPLS3 force field. The maximum number of stereoisomers produced for the ligand was 32, and this was determined by selecting the ionization states at pH 7.0 ± 2.0 (Aboul-Soud et al., 2022a; Slighoua et al., 2022).

To prepare the protein, the three-dimensional crystal structures of NADPH (PDB:2CDU), *S. aureus* nucleoside diphosphate kinase (PDB: 3Q8U), *E. coli* beta-ketoacyl-[acyl-carrier protein] synthase (PDB: 1FJ4), *Pseudomonas aeruginosa* LasR ligand-binding domain (PDB: 2UV0), and sterol 14-alpha demethylase (CYP51) from *C.* albicans (PDB: 5FSA) were obtained from the protein data bank in the PDB format. They were then built and refined using the Protein Preparation Wizard in Schrödinger Maestro v11.5. Hydrogen was added to the heavy atoms, selenomethionine was converted to methionine, and all water was removed. The protein was then minimized using the OPLS3 force field, with the maximum heavy atom RMSD set to 0.30 Å (Amrati et al., 2022; Aboul-Soud et al., 2022b).

The generation of the receptor grid involved launching the creation module by selecting a ligand atom, resulting in the creation of a default grid box. The ligand was then connected to the grid box generated from the protein using standard precision (SP).

SP flexible ligand docking was performed using the glide module of Schrödinger Maestro v 11.5, where penalties were imposed on non-cis/trans amide bonds. For the ligand atoms, the van der Waals scaling factor and partial charge cutoff were set at 0.80 and 0.15, respectively. The final score was determined using the energy-minimized poses and presented as a glide score. The best-docked pose for each ligand was recorded as the one with the lowest glide score value (Aboul-Soud et al., 2022a).

### 4.10 ADME/toxicity prediction

The prediction of ADME/toxicity was performed by analyzing the absorption, distribution, metabolism, and excretion parameters using the QikProp function in Maestro 11.5 of Schrödinger software. Various physicochemical and pharmacokinetic factors, such as molecular weight, hydrogen bond acceptor and donor, total solvent surface area, blood–brain partition coefficient, octanol/water partition coefficient, and aqueous solubility, were considered to support the hypothesis. Additionally, the ProTox-II server was utilized to investigate the organic toxicities and toxicological properties, including LD50, of the compounds identified in *T. broussonetii* EO (Banerjee et al., 2018).

### 4.11 Statistical analysis

Experiments were carried out in triplicate, and the values are expressed as the mean ± SD. Data were subjected to an analysis of variance (ANOVA). The level of significance was set at *p* < 0.05.

## Data Availability

The original contributions presented in the study are included in the article/supplementary materials; further inquiries can be directed to the corresponding author.
